# Combining global climate models using graph cuts

**DOI:** 10.1007/s00382-022-06213-4

**Published:** 2022-03-15

**Authors:** Soulivanh Thao, Mats Garvik, Gregoire Mariethoz, Mathieu Vrac

**Affiliations:** 1 Laboratoire des Sciences du Climat et l’Environnement (LSCE-IPSL) CNRS/CEA/UVSQ, UMR8212, Université Paris-Saclay, Gif-sur-Yvette, France; 2 Institute of Earth Surface Dynamics (IDYST), UNIL-Mouline, Geopolis, University of Lausanne, 1015 Lausanne, Switzerland

**Keywords:** Climate projections, Multi-model ensemble, Multi-model aggregation, Graph cuts

## Abstract

Global Climate Models are the main tools for climate projections. Since many models exist, it is common to use Multi-Model Ensembles to reduce biases and assess uncertainties in climate projections. Several approaches have been proposed to combine individual models and extract a robust signal from an ensemble. Among them, the Multi-Model Mean (MMM) is the most commonly used. Based on the assumption that the models are centered around the truth, it consists in averaging the ensemble, with the possibility of using equal weights for all models or to adjust weights to favor some models. In this paper, we propose a new alternative to reconstruct multi-decadal means of climate variables from a Multi-Model Ensemble, where the local performance of the models is taken into account. This is in contrast with MMM where a model has the same weight for all locations. Our approach is based on a computer vision method called graph cuts and consists in selecting for each grid point the most appropriate model, while at the same time considering the overall spatial consistency of the resulting field. The performance of the graph cuts approach is assessed based on two experiments: one where the ERA5 reanalyses are considered as the reference, and another involving a perfect model experiment where each model is in turn considered as the reference. We show that the graph cuts approach generally results in lower biases than other model combination approaches such as MMM, while at the same time preserving a similar level of spatial continuity.

**Supplementary Information:**

The online version contains supplementary material available at 10.1007/s00382-022-06213-4.

## Introduction

Global circulation models (GCMs) are key tools to project as robustly as possible the potential evolution of the climate, especially since human activities were established to be the main cause of global warming (Solomon et al. 2009). However, because of climate internal variability and structural model uncertainties, global or regional differences between climate models and observations or reanalyses can occur. Hence, one can wonder whether those observed differences can lead to additional uncertainties or even biases in the climate projections (Palmer and Stevens 2019).

Biases can be adjusted statistically and various methods exist to do so, ranging from relatively simple methods that only correct the mean, to more sophisticated ones correcting the whole distribution, potentially in multivariate contexts (e.g., see François et al. 2020, for a review and intercomparison). Although bias adjustment generally improves the realism of the climate simulations—at least in terms of the criteria used to perform the correction and over the calibration period—this can be sometimes at the expense of the physical realism of model outputs when some dependencies (intervariable, spatial or temporal depending on the data) are not taken into account. Hence, various adjustment techniques were recently developed to account for such dependencies (e.g., Cannon 2018; Vrac 2018; Robin et al. 2019; Vrac and Thao 2020). However, when bias corrected, the simulations still present distinct trends from one model to another on the calibration period and potentially even more distinct on future projection periods with different responses to climate change forcing scenarios. This means that bias correction does not remove all uncertainties and that there is a need to extract a robust signal of climate change by combining different climate models.

The most widely used approach so far to extract a robust signal among different models is to assemble those models into Multi-Model Ensembles (MMEs) and average them into multi-model means (MMM, see, e.g., Tebaldi and Knutti 2007; Knutti et al. 2010). These MMEs and MMMs are part of the Coupled Model Intercomparison Project or CMIPs (Dufresne et al. 2013), as an essential tool to manage climate-related risks for our societies (Kunreuther et al. 2013). Common approaches to assemble MMEs include model weighting, and selection of representative ensemble members (Cannon 2015; Sanderson et al. 2015). Equal weighting is the most commonly used and straightforward way of combining climate models (Weigel et al. 2010), but it does not account for model performance or interdependence. Non-equal-weighting methods are based on a search for optimal weights to improve the MMM result, such as Bayesian Model Averaging (Bhat et al. 2011; Kleiber et al. 2011; Olson et al. 2016) or Weighted Ensemble Averaging (Strobach and Bel 2020; Wanders and Wood 2016). Furthermore, climate models cannot be considered independent because they are often based on similar assumptions, parameterizations and computer codes. Therefore, agreement between models does not necessary mean convergence to a reliable projection (Abramowitz et al. 2019; Knutti et al. 2017; Rougier et al. 2013). While metrics of distance between models can be used to represent the wide range in the degree of similarity (or dissimilarity) between models, distances do not translate directly into a measure of independence (Abramowitz et al. 2019). As a consequence, weighting methods have been proposed that assign weights to models based not only on their performance, but also on their dependence with other models, often quantified as the difference (or distance) between models’ outputs (Lorenz et al. 2018). Some authors have proposed, as a pragmatic approach, a single set of weights for a given ensemble of models, which should yield reasonable overall performance while accounting for inter-model dependence (Sanderson et al. 2017).

The main uncertainties in model combination approaches are related to models themselves and also to the construction of the MME. Other methods, such as the Reliability Ensemble Average (REA) (Giorgi and Mearns 2002) weight models by taking into consideration biases and trends. However, uncertainties remain, linked to the many different scenarios, the model response uncertainty and the variability of the climate (Hawkins and Sutton 2009). The size of the MME also generates uncertainties: a combination based on a large ensemble can perform worse than with a small ensemble constructed with only good models (Knutti et al. 2010), and weighting methods can increase the number of models needed to construct a well-performing combination (Brunner et al. 2020; Merrifield et al. 2019). Furthermore, the weights given to a model are generally global (i.e., same weight for all grid points), meaning that even if a model can represent Europe temperature very well, it can be considered as poor overall and will not contribute to improving Europe temperature projection in the combination. As a result, a global weighting approach might represent this area worse than a model alone.

Thus far, the use of spatially non-uniform weights varying for each grid point has not been thoroughly considered in the literature on GCM combination. The consideration of local characteristics has mostly been taken into account in regional studies where an optimal number of models is selected for a given region of the globe (Ahmed et al. 2019; Dembélé et al. 2020, e.g.,), or by analyzing the performance of a weighed ensemble per sub-region (Brunner et al. 2019, 2020; CH2018 2018; Lorenz et al. 2018; Olson et al. 2016). However, this way of proceeding might be suboptimal as the region is defined first (e.g. Europe), then the weights are defined given this study area. There is, thus, a strong potential for improved model combination if the weights and the regionalization are co-optimized at the grid point level. Another aspect of model averaging techniques is that they invariably tend to smooth out the spatial patterns found in the individual models, despite the fact that these patterns often originate from actual physical processes.

Per-grid point model combination methods have been considered in scientific domains other than global climatology, such as in meteorology, where authors have shown that using spatially variable parameters of ensemble precipitation or wind forecast models leads to increased performance (Kleiber et al. 2011; Thorarinsdottir and Gneiting 2010), showing the promise of such approaches. In particular, geostatistical approaches have been shown to provide an appropriate set of tools to characterize the spatial structure and inter-variable dependence, and to take these aspects into account in statistical ensemble approaches, e.g. (Furrer et al. 2007; Sain and Cressie 2007; Gneiting 2014).

In this paper, we propose a model combination approach that improves the reproduction of observed climatological multi-decadal means, minimizes bias and maintains local spatial dependencies. It is based on a technique called graph cuts (GC), mainly used in computer vision (Kwatra et al. 2003; Boykov and Funka-Lea 2006; Salah et al. 2011) and geostatistics (Mariethoz and Caers 2014; Li et al. 2016) to assemble or reshape images by “stitching” other images in the best possible way. We call this approach GC-based patchworking. The quality of the model combination is evaluated by the visibility of the stiches: the less visible they are, the better the result is. In practice, this quality is represented by a cost function called energy in the Markov Random Fields literature (Szeliski et al. 2008). GC algorithms allow minimizing this energy. Model output fields can be seen as images where each grid point is a pixel. Therefore, we can use GC algorithms to combine outputs from different climate models so that the combination exhibits fewer biases than the individual models, while preserving the spatial dependencies locally. The result is an assemblage (i.e., patchwork) of the best models in terms of biases, while maintaining spatial consistency, i.e. minimizing stitches between model patches.

In this work, we compare our new GC-based patchworking method with the traditional MMM approach. The data used in this study, the GC algorithm and the design of experiments are described in Sect. 2. Results are detailed in Sect. 3. Finally, Sect. 4 is dedicated to discussions and conclusions.

## Data and methods

### Models and reanalysis data

The reference data used in this study are the reanalysis from the European Centre for Medium-Range Weather Forecasts (ECMWF) ERA5 (Hersbach 2016). Daily surface temperature (TAS, in K) and precipitation (PR, in mm/day) data have been extracted for the period 1979–2019 over the entire globe. This work is also based on the 20 CMIP5 models listed in Table 1. For each model, we extracted the same variables as in ERA5: TAS and PR. For the 1850–2005 period, data are extracted from the historical simulations and for the 2006–2100 period, from the projections made under the Representative Concentration Pathway 8.5 (RCP8.5). The RCP8.5 is one of the four greenhouse gas concentration trajectories considered by the Intergovernmental Panel on Climate Change in their Fifth Assessment Report. Among those four scenarios, RCP8.5 is the scenario leading to the largest warming at the end of the century with an increase of + 8.5 W/m$^2$ in terms of radiative forcing. Since the aim of this work is to reconstruct the multi-decadal average field of a given variable, the original data at the daily scale are averaged over a period of either 20 or 30 years depending on the experiments conducted in this paper (see Sect. 2.3 for more details). To make the comparison possible, the models and reanalyses are re-gridded onto a 1$^{\circ } \times 1^{\circ }$ latitude-longitude grid using bi-linear interpolation, which corresponds to 65160 grid cells.

**Table 1:** List of CMIP5 models and runs used

Institute	Model	Runs
BCC	bcc-csm1-1-m	r1i1p1
BNU	BNU-ESM	r1i1p1
CCCma	CanESM2	r1i1p1
CMCC	CMCC-CESM	r1i1p1
CNRM-CERFACS	CNRM-CM5	r1i1p1
CSIRO-BOM	ACCESS1-0	r1i1p1
CSIRO-QCCCE	CSIRO-Mk3-6-0	r1i1p1
FIO	FIO-ESM	r1i1p1
INM	inmcm4	r1i1p1
IPSL	IPSL-CM5A-LR	r1i1p1
MIROC	MIROC-ESM	r1i1p1
MOHC	HadGEM2-CC	r1i1p1
MPI-M	MPI-ESM-LR	r1i1p1
MRI	MRI-CGCM3	r1i1p1
NASA-GISS	GISS-E2-H	r1i1p1
NCAR	CCSM4	r1i1p1
NCC	NorESM1-M	r1i1p1
NIMR-KMA	HadGEM2-AO	r1i1p1
NOAA-GFDL	GFDL-CM3	r1i1p1
NSF-DOE-NCAR	CESM1-CAM5	r1i1p1

### Graph cuts for multi-model combination

In this work, we use the GC approach to combine an ensemble of GCMs and reconstruct multi-decadal averages of climate fields. Our aim is to obtain a combination that is closer to a given reference than any of the individual models. This is done by selecting, for each location (here, grid point), the value of one of the GCMs. The selection of a GCM at each grid point to build the new map is called a labeling in the graph cuts literature. The labeling $\mathbf{f}$ is chosen such that it minimizes a cost function called Energy in the Markov Random Fields literature (Li 2009). In our case, the energy is chosen to represent the mismatch between the reference and the constructed map, and also to favor labelings that are spatially homogeneous, in order to preserve as much as possible the physical continuity of the selected GCMs. Hence, the energy $E(\mathbf{f})$ is made of two terms, the data energy $E_{data}(\mathbf{f})$ and the smooth energy $E_{smooth}(\mathbf{f})$:

1 $$\begin{aligned} E(\mathbf{f}) = E_{data}(\mathbf{f}) + E_{smooth}(\mathbf{f}) \end{aligned}$$

where the labeling $\mathbf{f} = (f_p, p \in P)$ is a tuple and $f_p$ denotes the selected model for the grid point $p \in P$, the set of all grid points.

The data energy, $E_{data}(\mathbf{f})$, represents the bias between the GC result and the reference used. It is computed as the sum of the absolute bias over the set of all grid points *P*:

2 $$\begin{aligned} E_{data}(\mathbf{f}) = \sum _{p \in P} D(f_p) \end{aligned}$$

where $D(f_p)$ is the absolute bias at grid point *p* and is equal to $|X_p(f_p) - ref_p|$. In this expression, $X_p(f_p)$ denotes the value given by the model $f_p$ attributed at the grid point *p*. $ref_p$ denotes the value of the reference (for instance, ERA5) at the same grid point *p*.

The smooth energy, $E_{smooth}(\mathbf{f})$, represents the quality of the labeling in terms of spatial consistency, i.e., the fact that selecting a model for one grid point and another model for an adjacent grid point does not introduce a spatial discontinuity. This property will be referred to as “smoothness” hereafter:

3 $$\begin{aligned} E_{smooth}(\mathbf{f}) = \sum _{(p,q) \in N} V_{\left\{ p,q \right\} }(f_p,f_q). \end{aligned}$$

where *N* is the set of adjacent grid points and *p* and *q* represent two adjacent pixels. $V_{\left\{ p,q \right\} }$ is defined in the same way as the capacity cost in Li et al. (2016):

4 $$\begin{aligned} V_{\left\{ p,q \right\} }(f_p,f_q)= |X_p(f_p) - X_p(f_q)| + |X_q(f_p) - X_q(f_q)|. \end{aligned}$$

Note that when $f_p = f_q$, then $V_{\left\{ p,q \right\} }(f_p,f_q) = 0$. Furthermore, $V_{\left\{ p,q \right\} }(f_p,f_q) = 0$ if and only if $X_p(f_p) = X_p(f_q)$ and $X_q(f_p) = X_q(f_q)$. Hence, $V_{\left\{ p,q \right\} }(f_p,f_q) = 0$ means that the difference between two adjacent grid points is realistic since this difference is originally present in the two models $f_p$ and $f_q$.

**Fig. 1:**
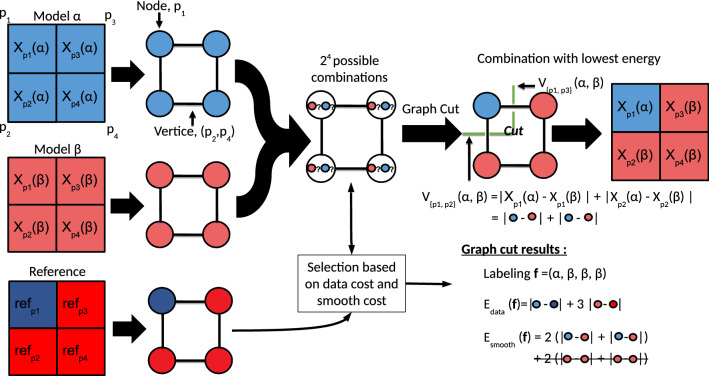
Illustration of the GC approach for 2 models. First, climate fields are represented as graphs where grid points are nodes and adjacency between grid points are vertices. Then, the GC algorithm finds the combination of models that minimizes the energy (data and smooth energy). Green dashed lines represent the “seams” made to combine the two models. Strike-trough terms in the smooth energy are equal to zero singe they do not corresponds to seams

**Fig. 2:**
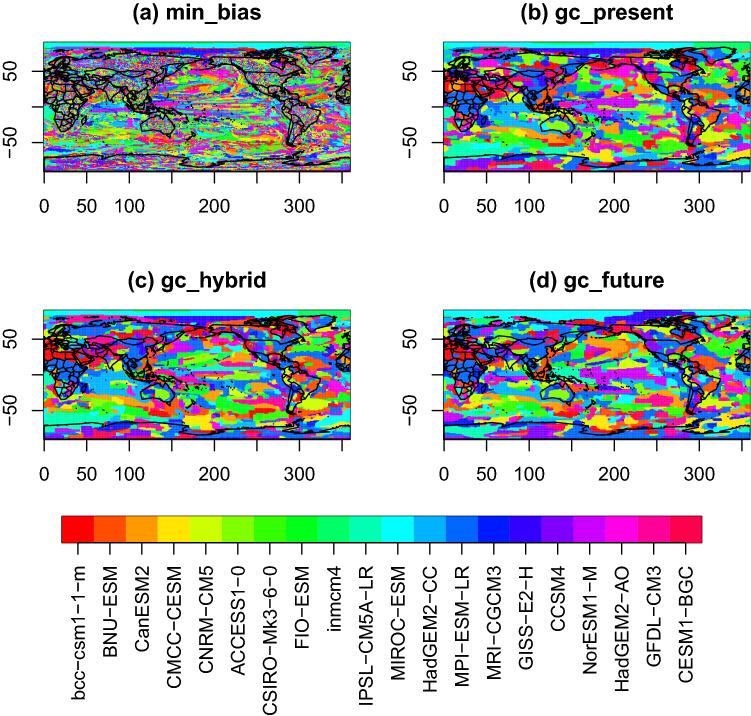
Maps of models selected at each grid point for the reconstruction of TAS in the ERA5 experiment. Each map represents the labeling obtained for one of the GC approach: **a** min_bias, **b** gc_present, **c** gc_hybrid, **d** gc_future

Figure 1 is a schematic illustration of the combination of two models ($\alpha$ and $\beta$) using the GC approach. In this figure, the reference and the models are represented as 2 by 2 matrices where each element represents a grid point, the value of which (e.g., mean temperature over 30 years) is represented by a color. Those matrices can also be represented as graphs where each grid point corresponds to a node (circle) and adjacent grid points are connected by a vertice (segment). In this setting with 4 grid points, there are $2^4$ possible combinations since each grid point can either be attributed the label $\alpha$ or $\beta$. The GC approach tries to find a combination of the two models that minimizes an energy function according two criteria: (1) the match to a reference (data energy) and (2) the spatial consistency of the combination (smooth energy). In the graph representation, the data energy is the sum of the costs associated with the nodes while the smooth energy is the sum of the costs associated with the vertices. The green dashed line represents the seams of the GC, that is the frontiers between selected models. Only the vertices crossed by the green dashed lines have an associated smooth energy greater than 0.

When the number of models to combine is equal to 2, a solution can be found through optimization by finding a global minimum of the energy function, resulting in an optimal labeling (Ishikawa 2012). In practice, we often have more than two models, e.g., 20 in the present study. In this case, we use an iterative approximation developed by Boykov et al. (2001): the $\upalpha$-$\upbeta$ swap algorithm. It starts by forming a solution with only one pair of models. Then one model in the pair is replaced by another and grid points attributed to either model in the pair are allowed to switch label: for a pair of models ($\alpha$, $\beta$), a grid point with the label $\alpha$, can have its label changed to $\beta$ if it reduces the energy *E*, and vice versa. This is repeated a number of times for all pairs of models until the energy *E* stops decreasing. Contrarily to the two-model case, this procedure only ensures that a local minimum of energy is reached. Hence, the whole procedure can be repeated a certain number of times with different initializations and orders of models and the outcome with the lowest energy can be retained. In practice, for our datasets and the few sensitivity tests we made, results were very similar (not shown) and the order of models in the $\upalpha$–$\upbeta$ swap algorithm did not matter much. For this reason, we have chosen in this paper to simply run the $\upalpha$–$\upbeta$ swap algorithm once and initialize it with the labeling that minimizes the data energy.

**Fig. 3:**
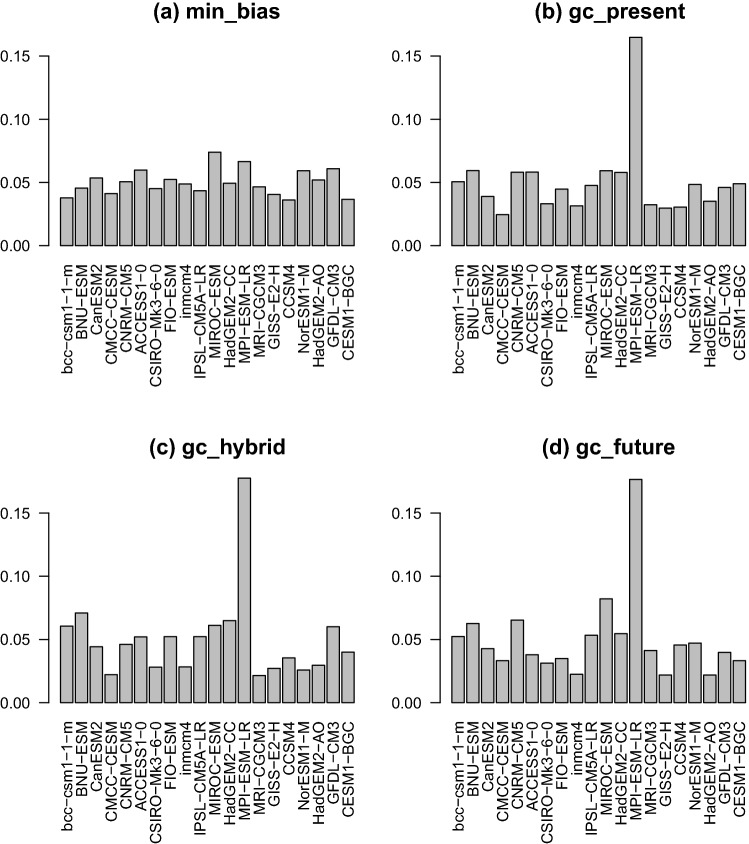
Histograms of the number of grid points attributed to each model for the different graph cut approaches used for the construction of TAS in the ERA5 experiment

**Fig. 4:**
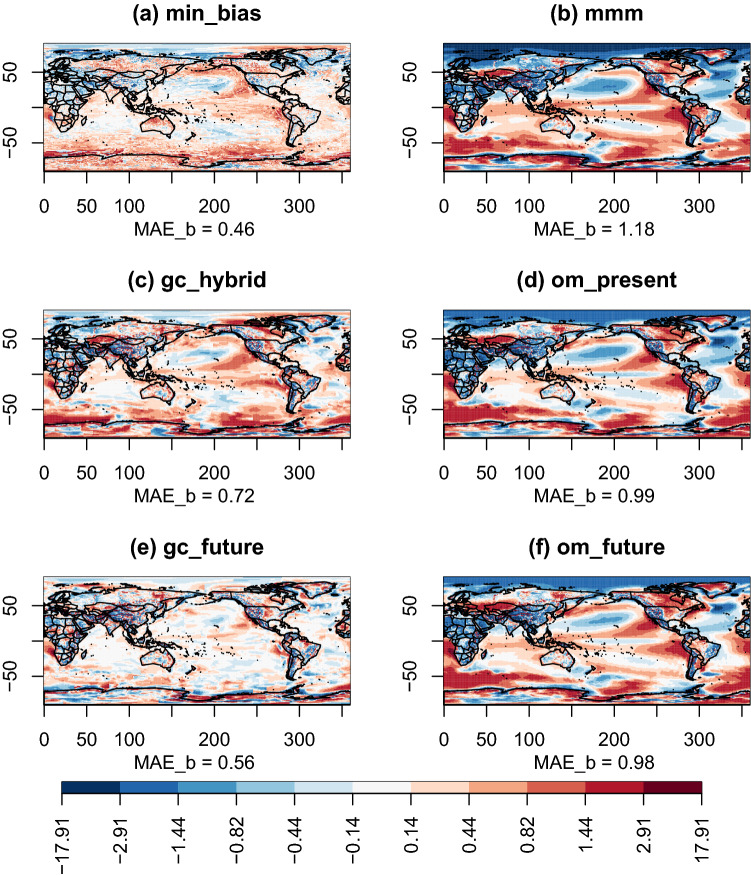
Maps of biases with respect to the reference ERA5 for the different combination approaches used to reconstruct the multi-decadal mean of TAS over the period 1999–2019. Note that the color scale is not linear (arctangent scale)

### Design of experiments

#### Combination approaches

In this paper, we compare the performance of different multi-model combination approaches, either based on MMMs or on GC. They are evaluated based on out-of-sample testing: when needed, the approaches are tuned on a calibration period (learning dataset) and their performances are evaluated on a projection period (test dataset). This way, the robustness and generalization capability of the combination approaches can be assessed. We have selected three approaches from the MMM family and four from the GC one:

- multi-model mean (mmm): each model is given the same weight to compute the average. Since it is the most commonly used approach in the literature, the multi-model is used as a baseline in this study.
- om_present: a weighted multi-model mean where the weight of each model is optimized on the calibration period in order to minimize the cost function:
  5 $$\begin{aligned} C(\mathbf{w}) = \sum _{p \in P} \left [ref_p - \sum _{f \in F} w_f X_p(f_p)\right ]^2 \end{aligned}$$
  where the weights $\mathbf{w} = (w_f)_{f \in F}$ are positive and sum up to 1. Note that the same weight is used for all grid points.
- om_future: same as om_present except that the models weights are optimized on the projection period. This aggregation method cannot be used in practice since the needed reference dataset in the projection period is unlikely to be available. It serves as a basis to assess the best results one could achieve in terms of bias with a multi-model mean approach (provided all information about the reference are available).
- min_bias: at each grid point, we select the value of the model having the smallest absolute bias in the calibration period. The same labeling is kept for the projection period. It corresponds to the result of a GC where only the data energy is minimized.
- gc_present: a GC procedure where the data energy and smooth energy are defined (and optimized) with respect to the calibration period.
- gc_future: a GC procedure where the data energy and the smooth energy are defined with respect to the projection period. Similarly to om_future, this aggregation cannot be used in practice since the reference dataset in the projection period needed for the data energy is unlikely to be available. However, gc_future gives an idea of the best results one could achieve with graph cuts.
- gc_hybrid: a GC procedure where the data energy is defined with respect to the calibration period and where the smooth energy is defined with respect to the projection period. This is possible in practice as the smooth energy only depends on the values of the models and not on the reference. The formulation of gc_hybrid can make more sense than the gc_present as we evaluate the degree of spatial continuity in the projection period and not in the calibration period.

#### Experiments

The evaluation of the combination approaches is performed based on two independent experiments:

1. An experiment where we use the ERA5 reanalysis data as reference. This experiment is quite realistic as reanalyses assimilate observations. It gives an indication about the combination performances when trying to reconstruct the true multi-decadal average field even if reanalyses are not exempt from uncertainties. The drawback of working with observations is that observational records are relatively short. Thus, the performances of the combination approaches are assessed on a projection period close in time to the calibration period. Consequently, the robustness of a combination approach to a strong evolution in the climate can be difficult to deduce from this experiment. In this case, the calibration period is defined as 1979–1998 and the projection period as 1999–2019. Hence, changes in the multi-decadal average fields between the two periods are likely to be relatively small.
2. An idealized perfect model experiment where we select one model as a reference that we try to reconstruct with the other models. In particular, this allows us to test the robustness of the different combination approaches under climate change. Here, the different combination approaches are calibrated on the historical period 1979–2008 and evaluated on a future period 2071–2100 as projected by the RCP8.5 scenario where there is an important warming. Although we do not use observational data as reference, this experiment can be justified under the “models are statistically indistinguishable from the truth” paradigm. Indeed, in this paradigm, the truth and the models are supposed to be generated from the same underlying probability distribution (e.g., Ribes et al. 2016). This means that the role of “truth” and a “model” can be exchanged without modifying the underlying probability distribution. Hence, an approach based on the “models are statistically indistinguishable from the truth” paradigm should also work when any model is considered as the reference. In our experiment, each model is used once as a reference, for both for calibration and projection. Note that ERA5 reanalysis is not used in this experiment. The combination approaches are thus tested on a variety of possible references, encompassing cases where the truth is either in the center of the multi-model distribution or far in the tail.

The ERA5 experiment assesses the performance of the combinations approaches on very short-term projections where the main source of uncertainty is the internal variability of the climate. Contrastingly, the perfect model experiment assesses the performance of long-term projections where the main uncertainties are related to the multi-model spread in the climate projections.

**Fig. 5:**
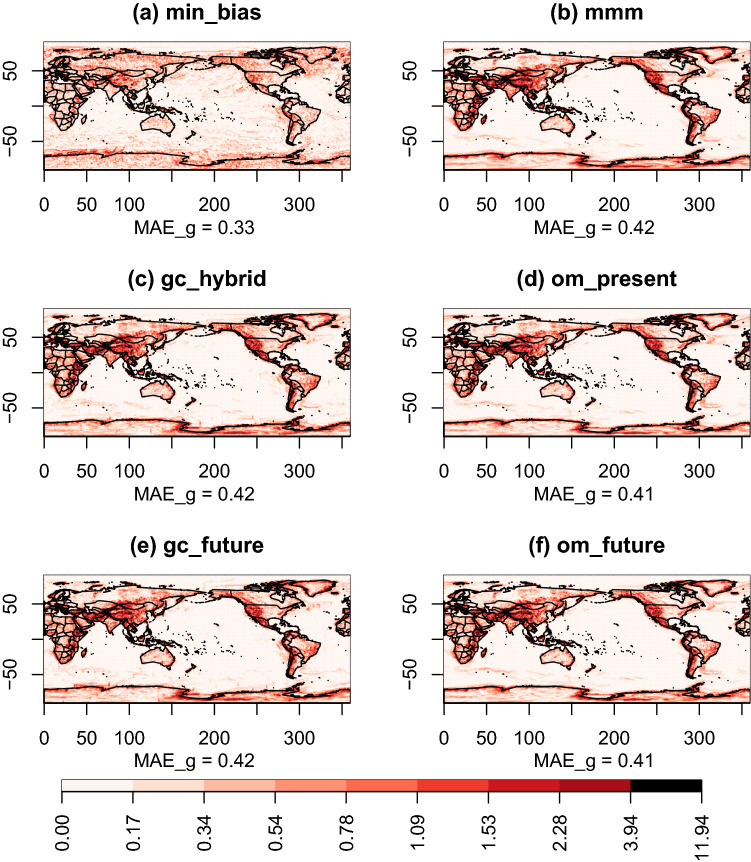
Maps of $MAE_{g}^{(p)}$ with respect to the reference ERA5 for the different combination approaches used to reconstruct the multi-decadal mean of TAS over the period 1999–2019. Note that the color scale is not linear (arctangent scale)

**Fig. 6:**
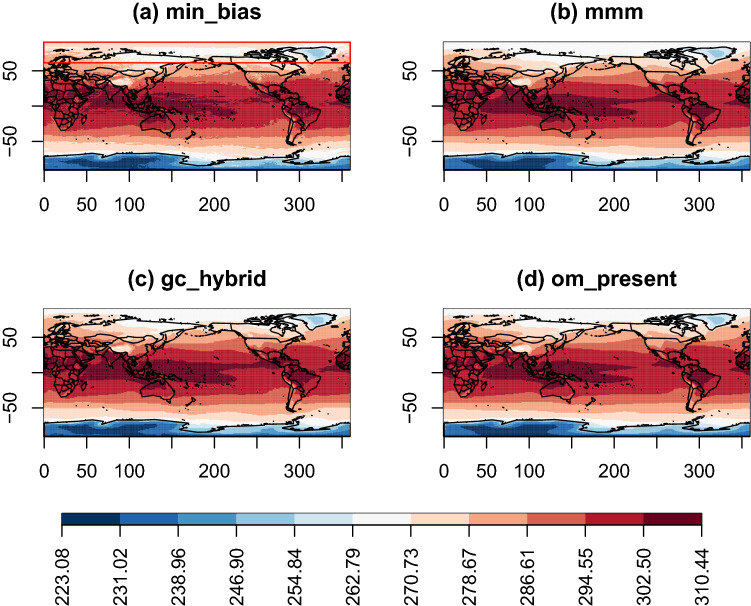
Maps of the projected multi-decadal mean of the variable TAS over the period 2071–2100. They are obtained for the ERA5 experiment with the following combination approaches: **a** min_bias, **b** mmm, **c** gc_hybrid, **d** om_present. The red rectangle highlights the region in the min_bias projection where the usual latitudinal gradient of temperature is not reproduced

**Table 2:** Performance metrics of the different combination approaches used to reconstruct the multidecadal mean of PR during the period 2000–2019

Approach	$MAE_{b}$	$MAE_{g}$
mmm	0.46	0.18
om_present	0.39	0.17
om_future	0.38	0.17
min_bias	0.22	0.16
gc_present	0.32	0.18
gc_hybrid	0.32	0.18
gc_future	0.23	0.17

**Fig. 7:**
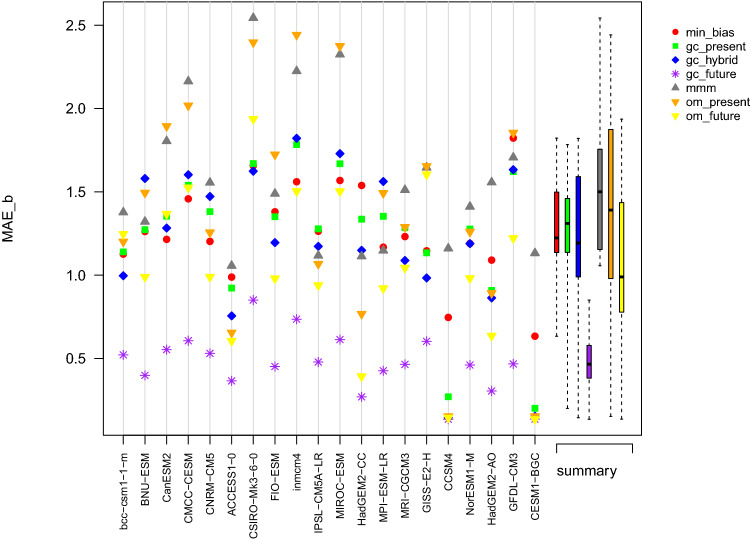
Summary plot of the $MAE_b$ obtained in the perfect model experiment for the variable TAS and computed over the projection period 2071–2100. The abscissa axis indicates the model used as reference

**Fig. 8:**
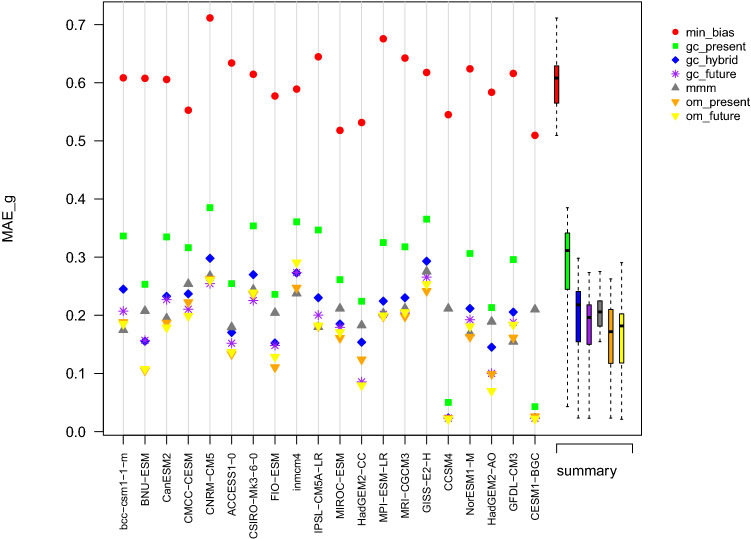
Summary plot of the $MAE_g$ obtained in the perfect model experiment for the variable TAS computed over the projection period 2071–2100. The abscissa axis indicates the model used as reference

**Fig. 9:**
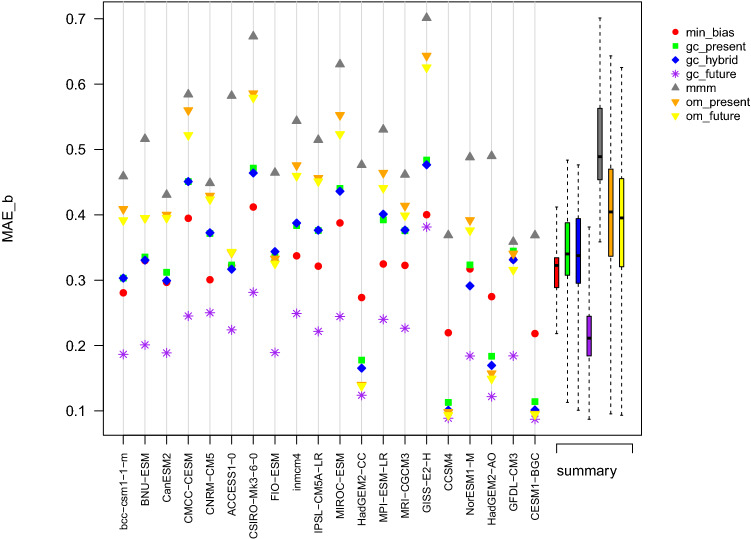
Summary plot of the $MAE_b$ obtained in the perfect model experiment for the variable PR and computed over the projection period 2071–2100. The abscissa axis indicates the model used as reference

**Fig. 10:**
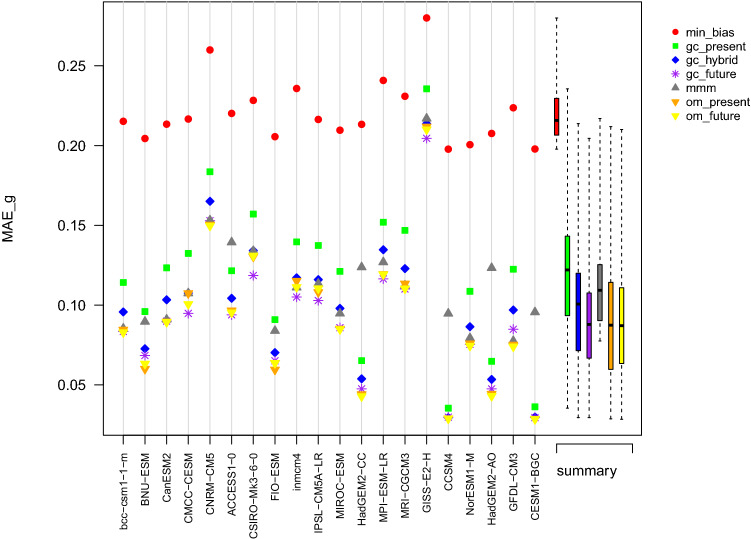
Summary plot of the $MAE_g$ obtained in the perfect model experiment for the variable PR computed over the projection period 2071–2100. The abscissa axis indicates the model used as reference

#### Evaluation metrics

In both experiments, the combination approaches are evaluated on two aspects, the biases and the spatial gradients:

1. The biases reflect the local error of a combination approach with respect to the reference *ref*, quantified by the mean absolute error (MAE). It is calculated by averaging the absolute value of the bias at each grid point:
  6 $$\begin{aligned} MAE_{b}(\mathbf{f}) = \frac{1}{\#P}\sum _{p \in P} \big |X_p(f_p) - ref_p\big | \end{aligned}$$
  where $\#$ denotes the cardinal number of a set. Note that, for a given GC combination, $MAE_{b}$ is simply the data cost on the projection period normalized by the number of grid points $\#P$.
2. A spatial gradient is defined as the difference of values between one grid point and one of the adjacent grid cell. The spatial gradients are used to determine whether the combination approaches represent well the spatial distribution of the reference. Indeed, GC approaches can introduce spatial discontinuities since their results are a patchwork of models. Additionally, MMM approaches can be expected, by construction, to have smoother results, and thus gradients smoother than the reference. Overall, the ability of the approaches to reproduce the spatial gradients of the reference is evaluated in terms of mean absolute error (MAE):
  7 $$\begin{aligned} MAE_{g}(\mathbf{f}) = \frac{1}{\#P}\sum _{p \in P} MAE_{g}^{(p)} \end{aligned}$$
  where:
  8 $$\begin{aligned} MAE_{g}^{(p)} = \frac{1}{\#N_p} \sum _{q \in N_p} \big |\big (X_p(f_p) - X_q(f_q)\big ) - \big (ref_p - ref_q\big )\big | \end{aligned}$$
  and $N_p$ denotes the grid points adjacent to the grid point *p*. Note that $MAE_{g}$ is not independent of $MAE_{b}$. When $MAE_{b}(\mathbf{f}) = 0$, then $MAE_{g}(\mathbf{f}) = 0$.
  Note that, in the graph cut approach, the smooth energy reflects the level of discontinuity in the resulting combination. However, this metric is a function of the selected labels and thus, it can only be used for graph cut based approaches. This is why we evaluate the spatial variability of the different combinations with the spatial gradients instead. The spatial gradients characterize the spatial variability between one grid point and its neighbor. Hence, spatial gradients only represent the local spatial structure. Nevertheless, they make sense in the context of the graph cut approaches where the spatial discontinuity is only defined in the smooth cost with respect to one grid point and its neighbors.

## Results

### ERA5 experiment

In this section, we examine the performance of the various combination approaches in reconstructing the 1999–2019 multi-decadal average of ERA5 surface temperature (TAS, in K) and total precipitation (PR, in mm/day). For conciseness, we will thoroughly present the results for TAS and only point out notable results for PR. The performance is evaluated in terms of biases and spatial gradients. As a reminder, all multi-model approaches except gc_future and om_future are calibrated during the period 1979–1998 and evaluated during the period 1999-2019.

#### Reconstruction of TAS

Figure 2 shows the labeling obtained for the four graph cuts approaches. gc_present, gc_hybrid and gc_future show very similar labelings. This can be explained by the fact that, for all models and for the reference, the multi-decadal average of the TAS fields does not change much from 1979–1998 to 1999–2019. The labeling obtained with min_bias is noisier, with significant variability in the labels between adjacent grid points. However, the histogram of labels used is more uniform than in the other GC approaches (Fig. 3). For instance, for gc_present, gc_hybrid and gc_future, MPI-ESM-LR is the most used model and is attributed to more than 15% of the grid points. For min_bias, each model is attributed to about 5% of the grid points. It suggests that all models have some value when considering only the bias at the grid point scale: for each model, there is a grid point where the absolute bias with respect to the reference is the minimum.

It is noted that gc_present is not informed by climate projections, therefore it is not deemed relevant for practical purposes. Hence, in the following (including in the perfect model experiment), we will not present further results in terms of maps for gc_present, especially as gc_present is similar to gc_hybrid in terms of biases and is most of the time between min_bias and gc_hybrid in terms of spatial gradients (not shown).

All approaches show similar structures of biases (Fig. 4). In general, we observe negative biases over the Arctic Ocean and over Africa and positive biases over Antarctica, the Southern Ocean and upwelling areas. The differences between the approaches are more related to the intensity of the biases than to their spatial structure. The MMM-based approaches (mmm, om_present and om_future) perform poorest ($MAE_{b}$ of 1.18, 0.99 and 0.98, respectively). The results for om_future show that using a global weight for each model is not sufficient to reconstruct the local distribution of temperature. gc_present and gc_hybrid have similar performance ($MAE_{b}$ of 0.71 and 0.72). gc_future has the second best result ($MAE_{b}$=0.56) behind min_bias ($MAE_{b}$=0.46). This can be surprising as gc_future has been calibrated on the projection period, but it probably suggests that the bias with the reference does not change much between the calibration and projection period. Note that in gc_future, a compromise is made between the data energy and the smooth energy which can also explain why it is not performing as well as min_bias that only considers the biases. Out of all approaches, min_bias is the approach with the noisiest spatial pattern of bias, which is expected as it does not consider spatial continuity.

In terms of spatial gradients, all approaches exhibit similar patterns of differences with the reference (Fig. 5). Strong disparities with the reference are located in continental areas, in particular in regions with high reliefs. The main difference between the approaches is the intensity of these differences. All approaches except min_bias show similar performance ($MAE_g \sim$ 0.42). min_bias has the best performance by quite a large margin ($MAE_g$ = 0.33). For min_bias, the pattern of discrepancies is noisy, with a large number of grid points having $MAE_{g}^{(p)}$ close to zero. Contrary to others approaches, there are differences in the spatial gradients in the oceans, but their intensities are low. To visualize the statistical distributions of the biases and the gradient errors, Figs. 4 and 5 are respectively represented as histograms in Fig. S1 and Fig. S2.

It is worth noting at this point that good results on the period 1999–2019 do not imply that the projections at the end of the century are also of good quality. Hence, we look at the temperature projected for 2071–2100 by the different combination approaches, even though a quantitative assessment of the projections cannot be made. Indeed, the ERA5 reanalysis, which serve as reference, are not available for this period. Nonetheless, we can observe that while the patterns of temperature projected for 2071–2100 are quite similar among the different approaches (Fig. 6), only gc_present and min_bias do not fully respect the latitudinal gradient of temperatures and exhibit temperatures at 90 degrees north being higher than at 70 degrees north, which seems non-physical. Indeed, no projections made with individual models show such a pattern (not shown). Hence, even though min_bias shows the best results both in terms of both bias and spatial gradient for 1999–2019, projections made with the min_bias approach for end of the century can lack robustness. The constraint brought by the smooth energy appears to help producing more robust projections. Other differences between the combination approaches occur near the Intertropical Convergence Zone (ITCZ). In this region, gc_hybrid is closer to mmm and min_bias is closer to om_present.

#### Reconstruction of PR

Similar conclusions can be reached for the reconstruction of PR. The spatial patterns of biases and errors in the gradients are similar among the different approaches (Fig. S3, Fig. S4, and Fig. S5, Fig. S6 for the corresponding histograms.) Errors in terms of biases and spatial gradients are more important around the ITCZ. In this region, discrepancies in the gradients appear at the boundary between regions of negative and positive biases. In terms of spatial gradients, all methods have similar performance but in terms of bias, GC approaches exhibit better results, especially min_bias (Table 2). For the projections at the end of the 21st century, mmm exhibits an increase in precipitation near the ITCZ whereas other methods show more nuanced patterns with a few regions in the ITCZ where precipitation decreases (Fig. S7).

### Perfect model experiment

In this section, we present the results of the perfect model experiment. Since for a given reference, the evaluation procedure is the same as the one employed in the ERA5 experiment, we will only present the results summarized over all reference models. As for the ERA5 experiment, the combination approaches are evaluated for TAS and PR in terms of biases and spatial gradients. As a reminder, all combination approaches except gc_future and om_future are calibrated on the period 1979–2008 and evaluated on the period 2071–2100.

#### Summary of TAS reconstruction

Here we examine the results obtained once every model has been used as a reference for the variable TAS. Results in terms of biases are summarized in Fig. 7. Depending on the reference, the performance of the different approaches in terms of $MAE_b$ varies substantially. Additionally, from one reference to another, the ranking of the approaches can be quite different; we can however distinguish trends. For all references, gc_future has the best performance, often by a large margin: this is expected since it is calibrated on the projection period. The second best performance is achieved by om_future, which is also calibrated on the projection period. The gap between gc_future and om_future shows that having one unique and global weight per model is sometime not enough to reconstruct the multi-decadal mean temperature. It is also interesting to note that when CCSM4 or CESM1-BGC are used as reference, om_present and om_future reach the same level of performance, and gc_hybrid is not too far behind. However, the results of om_present highly depend on the reference. On average, the worst results are obtained with mmm. The graph cuts approaches, min_bias, gc_present and gc_hybrid, tend to perform similarly. The median of the $MAE_b$ is slightly better for gc_hybrid, but the variability of $MAE_b$ is higher than for min_bias and gc_present. Over all references and on average, the combination approaches have more difficulties estimating the temperature multi-decadal average in the Arctic Ocean and on the continents (Fig. S8).

Results in terms of spatial gradient are summarized in Fig. 8. The worst results are obtained with the min_bias approach, as expected since there is no constraint on the spatial consistency in the labeling selection. The second worst results are obtained by gc_present. It is understandable since the smooth energy is not optimized on the projection period. The five remaining approaches have comparable performances. In average, there is a slight advantage for om_present and om_future. There are cases where om_present performs better in terms of spatial gradient than om_future. It can be explained by the fact that even if om_future is calibrated on the projection period, the weights are chosen to only minimize the bias without accounting for the spatial gradients. Hence, there are cases when minimizing the bias degrades the spatial gradients. gc_future is only the third best approach despite being calibrated directly on the projection period, and despite using the knowledge of the reference in the future. It suggests that for very smooth fields such as the multi-decadal mean of TAS, patching models together incurs a loss in terms of spatial gradient compared to MMM approaches, especially if the spatial gradients are already well represented in the individuals models. Over all references and on average, the spatial gradients in mountainous regions are not well reproduced by any of the combination approaches (Fig. S9). This suggests that the models exhibit large discrepancies in those areas. Those are also the areas where gc_hybrid, gc_future, and om_present show small improvements compared to mmm.

When looking at the maps of temperature produced for the end of the century projections, min_bias and gc_hybrid can show spatial patterns that appear unrealistic, depending on the reference. In the case of min_bias, as in the ERA5 experiment, the usual meridional gradient of temperatures is sometimes not fully respected in high latitudes (not shown). For gc_hybrid, in a few cases, we can clearly distinguish the seams of the patchworks made by the GC algorithm in high latitudes. It usually corresponds to one model that has been attributed to one large area and exhibits values quite different from the other selected models (not shown).

#### Summary of PR reconstruction

For PR, results are similar to TAS in terms of bias in the sense that GC approaches (with the exception of min_bias) tend to have smaller biases than comparable MMM approaches (Fig. 9). The difference in bias is however clearer than for temperature since all GC approaches give better results than om_future. In terms of spatial gradients, om_present and om_futur give slightly better results than gc_hybrid and gc_future (Fig. 10). As in the ERA5 experiment, all methods have difficulties reconstructing the region of the ITCZ, both in terms of biases (Fig. S10) and of spatial gradients(Fig. S11).

## Conclusions and discussion

In this paper, we introduced the Graph Cuts (GC) algorithm (e.g., Kwatra et al. 2003; Boykov and Funka-Lea 2006) as an alternative to multi-model means (MMM) to extract the robust signal of climate change in a multi-model ensemble. The GC was used to estimate the multi-decadal mean field of a climate variable. GC approaches distinguish themselves from the traditional MMM based approaches that are widely used in the literature. Indeed, the GC approaches construct their estimations by selecting at each grid-cell the value of the ensemble member that is considered the best, i.e., the member that minimizes the bias and maximizes spatial consistency. Hence, it can be seen as a particular case of a MMM approach using local weights for the models. In the case of the graph cuts, the weight of a given model at a given location is simply either equal to 1 or 0.

We have evaluated the ability of GC approaches to predict the multi-decadal mean of a climate field (TAS or PR). The performances of GC approaches were compared to three MMM approaches with global weights: mmm where each model has the same weight; om_present and om_future where the weights of each model are respectively calibrated based on the model biases in the calibration period and projection period.

Performances were assessed based on two experiments: one using ERA5 reanalyses as the reference and another one based on a perfect model experiment setting. The results of the ERA5 experiment showed that when the climate does not evolve much between the calibration and projection periods, GC approaches perform better in terms of biases and have a similar performance to mmm in terms of spatial gradients. In this experiment, the best results were obtained by far by the min_bias approach, both in terms of bias and spatial gradients. This approach simply selects, for each grid point, the value of the model with the minimum bias in the calibration period. We explain the good performance of min_bias by the fact that the climate can be considered almost stationary between periods 1979–1998 and 1999–2019. When the labeling given by min_bias is used for long term projections (2071–2100), it can lead to non-physical results. In the case of temperature, the latitudinal gradients of temperature are for instance not totally reproduced. Hence, this experiment did not allow us to assess the usability of the GC approaches for long-term projections.

Long-term projections were assessed with a perfect-model experiment, where all models were in turn used as reference. Results for TAS and PR showed that the best GC approach usable in practice is gc_hybrid. Compared to min_bias, the spatial consistency constraint brought by the smooth energy significantly improves the robustness of gc_hybrid. In general, the biases are more consistently reduced with gc_hybrid than with mmm. Depending on the reference selected, results of om_present were sometimes better than the gc_hybrid, but were sometimes the worst of all methods. The performance of om_present is thus less consistent. However, the gain obtained by gc_hybrid in terms of bias is also associated with a small loss in terms of spatial gradients compared to om_present. The comparison of om_future with gc_future shows that having only one global weight per model is not flexible enough to reconstruct the multi-decadal average of a field.

In both experiments, the GC results showed that every model was used in the reconstruction of the multi-decadal mean field. It indicates that every model can bring a meaningful contribution to some regions where its bias is lower than that of other models. Overall, our results show that GC based approaches provide an interesting way of using MME and are complementary to MMM approaches.

The results of these two experiments were evaluated based on two metrics: one related to the bias at each grid point and the other based on the error in the spatial gradients. The spatial gradients only look at the relationship between one grid point and its neighbors, so it is a very local metric. While we think that the two metrics used in this paper were sufficiently convincing to show the potential of the GC approach, the analysis of the results could be refined by using complementary metrics to assess how well the spatial variability is reproduced by the different combination approaches. For instance, the connectivity analysis (Renard and Allard 2013) and the fractions skill scores (Roberts and Lean 2008) are complementary metrics that look at the spatial variability at different spatial scales.

The GC approaches were introduced in this paper mainly as a proof of concept and could benefit from several improvements:

- One of the most important improvements would be to associate a degree of confidence or uncertainty to the reconstructed maps. This work would require additional hypotheses and to develop further the underlying statistical formulation of the GC approaches.
- When determining the labels in the GC approaches, the bias (data energy) and spatial consistency (smooth energy) have the same weight in the energy function. The performance of the GC approaches could be further improved if these weights could be optimally select. In the same idea, depending on the objectives when applying a model combination with a GC approach, such weights can be arbitrarily fixed: a practitioner more interested in preserving a spatial smoothness of the results than in the bias minimization would give a higher weight to the smooth energy than to the data energy, and conversely.
- In this paper, we observed that the labeling obtained for TAS and PR are different. To make consistent projections across different variables, the energy function could be defined such that the multi-decadal mean of TAS and PR are reconstructed together, resulting in a single labeling. More generally, the GC approach could be applied in a multivariate way, i.e., to more than one variable at the same time.
- Here, we run the GC algorithm on 2D maps without using the spherical geometry of the Earth. In particular, neighborhoods of grid points across the Greenwich meridian or across the poles are not considered. Additionally, in the GC procedures and in our evaluations, all grid points have the same weight despite covering different areas. This will need to be addressed in future implementations.
- We applied the GC approaches directly to model outputs. Before using GC approaches, model simulations could first be bias-corrected. Assessing the influence of bias correction on the multi-model combination approaches could be an interesting line of research.
- While we only demonstrated the GC approaches based on multi-decadal means, the applicability of the method should be tested other statistics (e.g., variance, extremes, etc.) or on different integration periods, such as to produce seasonal maps.
- In the same line of idea, one could use the graph cut to generate time-series or spatio-temporal data by combining slices of temporal sequences coming from different climate models. In this case, the graph cut would be used to generate additional realizations of a time series, which is distinct from the goal pursued in this paper to provide more robust climate projections. However, some challenges may arise from the internal climate variability. Indeed, since the climate system is chaotic, the outcome of different simulations are not synchronized and hence not correlated, neither between each other nor with the observations. This internal variability could be dealt with either by redesigning the data and smooth energies to account for the temporal variability, or to aggregate the data on long (e.g. decadal) time periods where the chaotic behavior is smoothed out. An application with spatio-temporal data, with the same objective as in this paper, would consist of working with statistics that are functions of space and time and computed on multi-decadal periods to reduce the effect of internal variability. For instance, one could use the graph cut approach to estimate the average seasonal cycle at the daily time scale and to ensure that there is a smooth transition between successive seasonal maps.

To conclude, GC is a promising method for applications to climate models combination, which we only start exploring in this paper.

## Supplementary Information

Below is the link to the electronic supplementary material.

Supplementary file1 (PDF 9982 KB)

## Data Availability

CMIP5 climate model simulations can be downloaded through the Earth System Grid Federation portals. Instructions to access the data are available here: https://pcmdi.llnl.gov/mips/cmip5/data-access-getting-started.html, last access: 08 February 2021, (PCMDI, 1989). The ERA5 reanalyses can be downloaded through the Climate Data Store (Hersbach et al. 2019). Last accessed: access: 08 February 2021.

## Declarations

### Code availability

The multi-label optimization with graph cuts is done with the c++ library gco. It is based on the following papers (Boykov et al. 2001; Kolmogorov and Zabin 2004; Boykov and Kolmogorov 2004) and is available here: https://vision.cs.uwaterloo.ca/code/ A simple wrapper around this library is publicly available for the R language at: https://github.com/thaos/gcoWrapR. R scripts to reproduce the analysis are available here: https://github.com/thaos/GraphCut_MMM
