# Supplementary material for: Combining global climate models using graph cuts
Source: Clim Dyn. 2022 Mar 15;59(7-8):2345–61. doi: 10.1007/s00382-022-06213-4 (PMC9463255; doi:10.1007/s00382-022-06213-4)
Supplement: Supplementary file 1 — Supplementary file1 (PDF 9982 KB) [file 382_2022_6213_MOESM1_ESM.pdf]

Climate Dynamics manuscript No.
(will be inserted by the editor)

Combining global climate models using graph cuts

**Soulivanh Thao · Mats Garvik ·
Gregoire Mariethoz · Mathieu Vrac**

Received: date / Accepted: date

Soulivanh Thao
Laboratoire des Sciences du Climat et l'Environnement (LSCE-IPSL) CNRS/CEA/UVSQ, UMR8212, Université Paris-Saclay, Gif-sur-Yvette, France
Tel.: +33-1690863197
email: sthao@lsce.ipsl.fr

Mats Garvik
Laboratoire des Sciences du Climat et l'Environnement (LSCE-IPSL) CNRS/CEA/UVSQ, UMR8212, Université Paris-Saclay, Gif-sur-Yvette, France

Gregoire Mariethoz
University of Lausanne, Institute of Earth Surface Dynamics (IDYST), UNIL-Mouline, Geopolis, 1015 Lausanne, Switzerland

Mathieu Vrac
Laboratoire des Sciences du Climat et l'Environnement (LSCE-IPSL) CNRS/CEA/UVSQ, UMR8212, Université Paris-Saclay, Gif-sur-Yvette, France

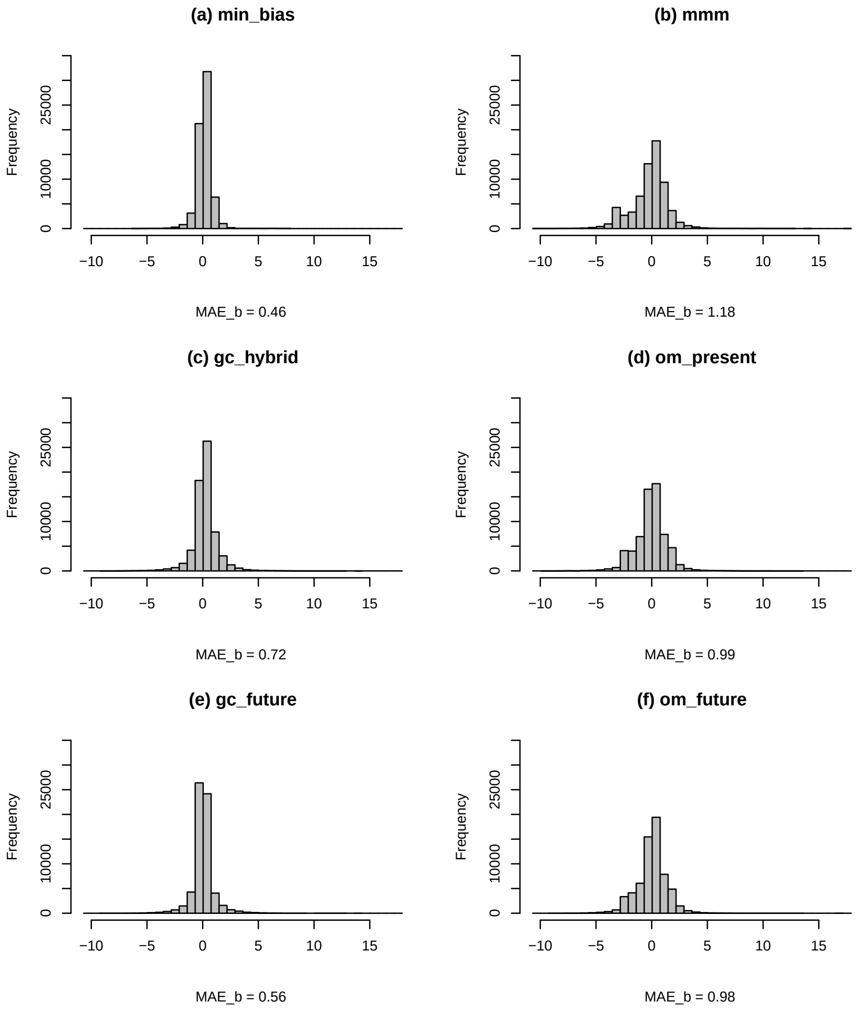

Fig. S1: Histograms of the biases at each grid-point with respect to the reference ERA5 for the different combination approaches used to reconstruct the multi-decadal mean of TAS over the period 1999-2019.

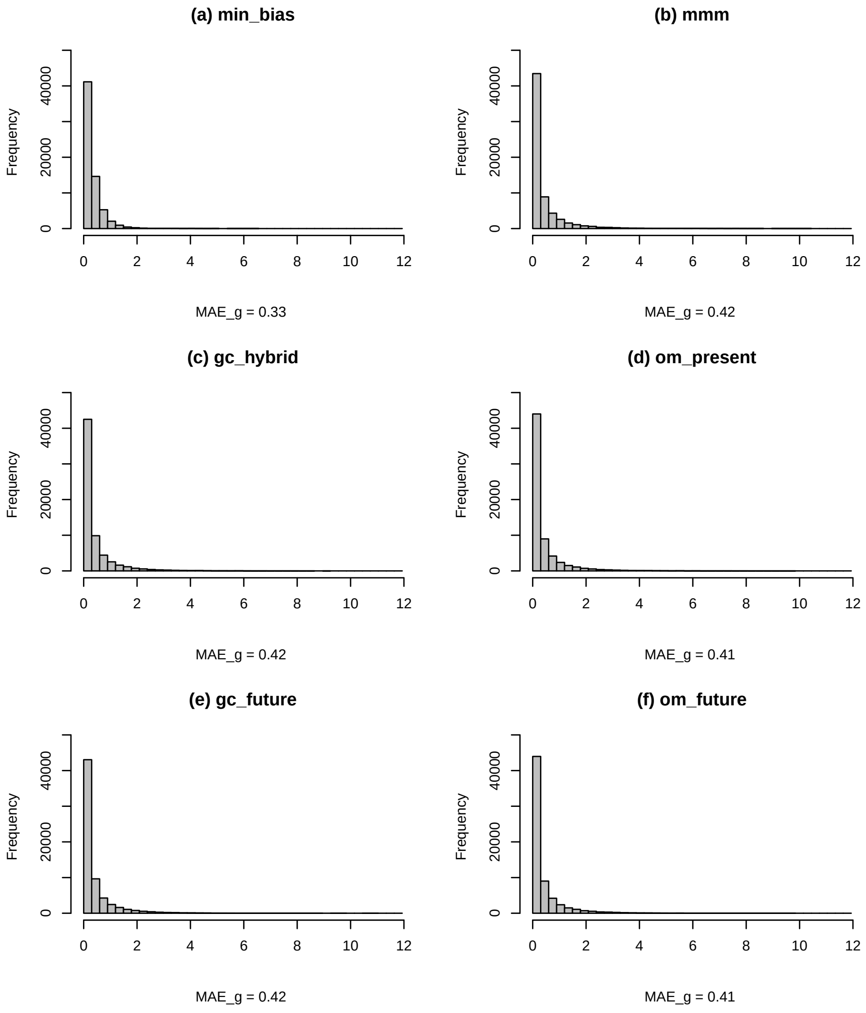

Fig. S2: Histograms of $MAE_g^{(p)}$ with respect to the reference ERA5 for the different combination approaches used to reconstruct the multi-decadal mean of TAS over the period 1999-2019

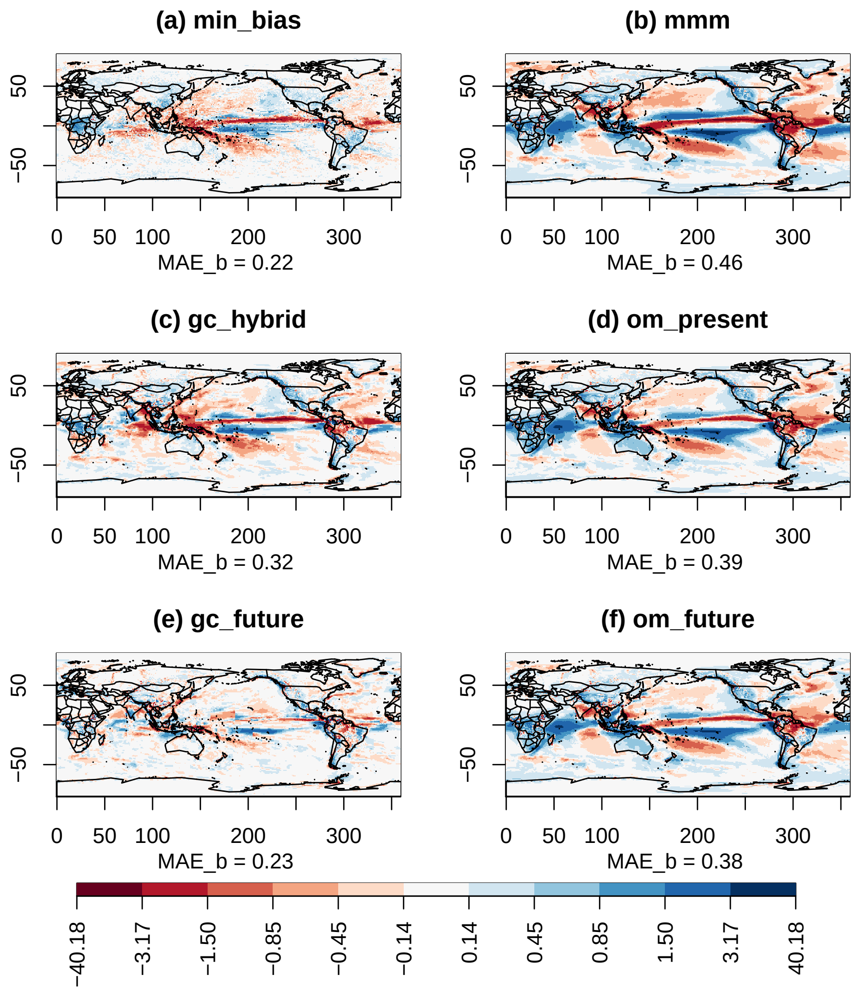

Fig. S3: Maps of biases with respect to the reference ERA5 for the different combination approaches used to reconstruct the multi-decadal mean of PR over the period 1999-2019. Note that the color scale is not linear (arctangent scale).

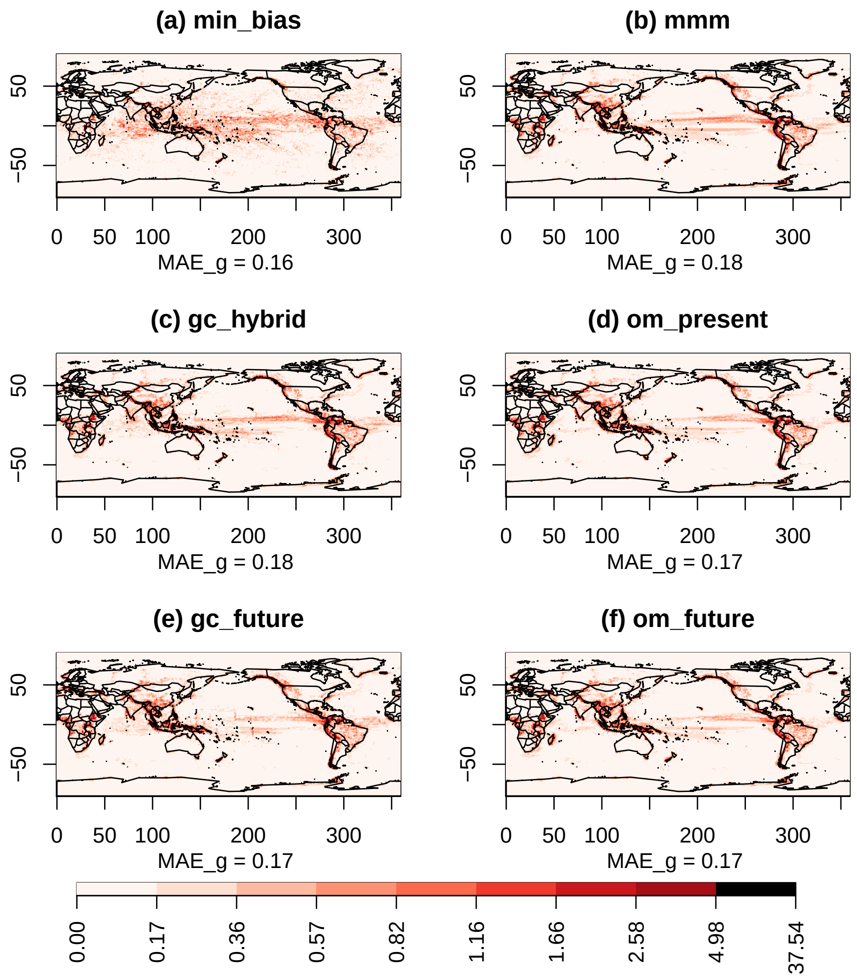

Fig. S4: Maps of $MAE_g^{(p)}$ with respect to the reference ERA5 for the different combination approaches used to reconstruct the multi-decadal mean of PR over the period 1999-2019. Note that the color scale is not linear (arctangent scale).

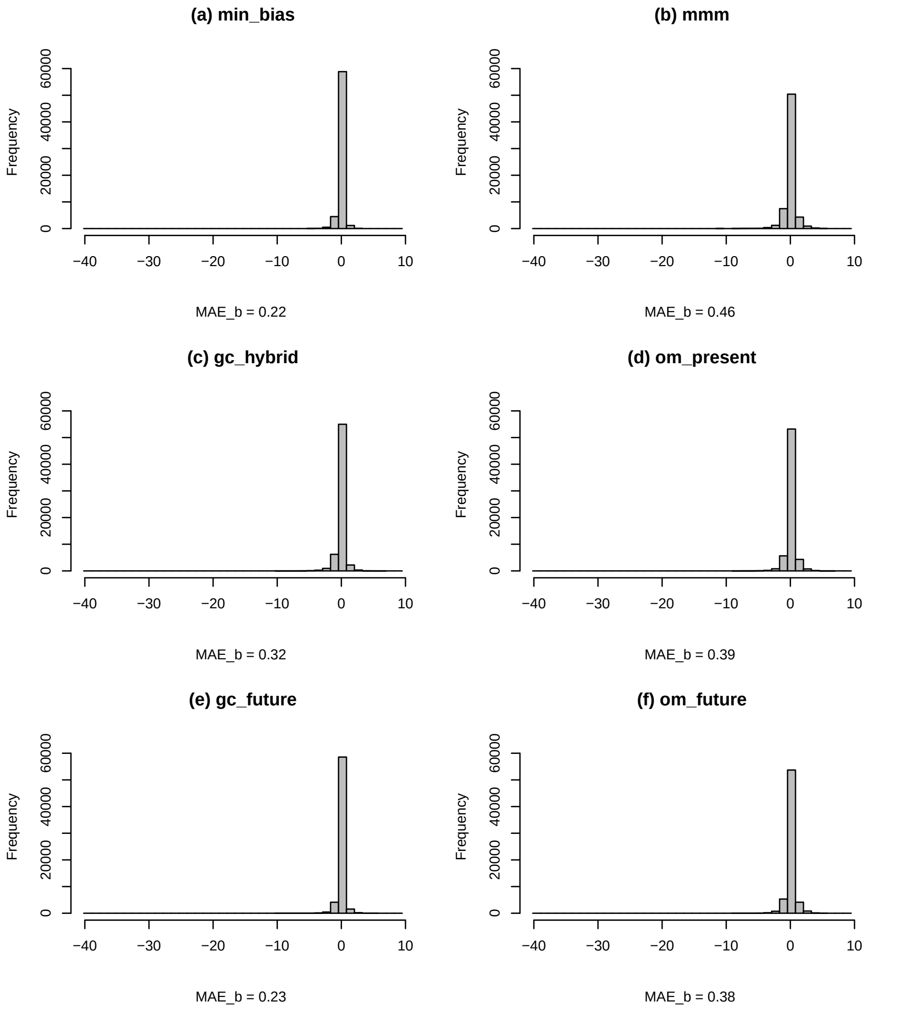

Fig. S5: Histograms of the biases at each grid-point with respect to the reference ERA5 for the different combination approaches used to reconstruct the multi-decadal mean of PR over the period 1999-2019.

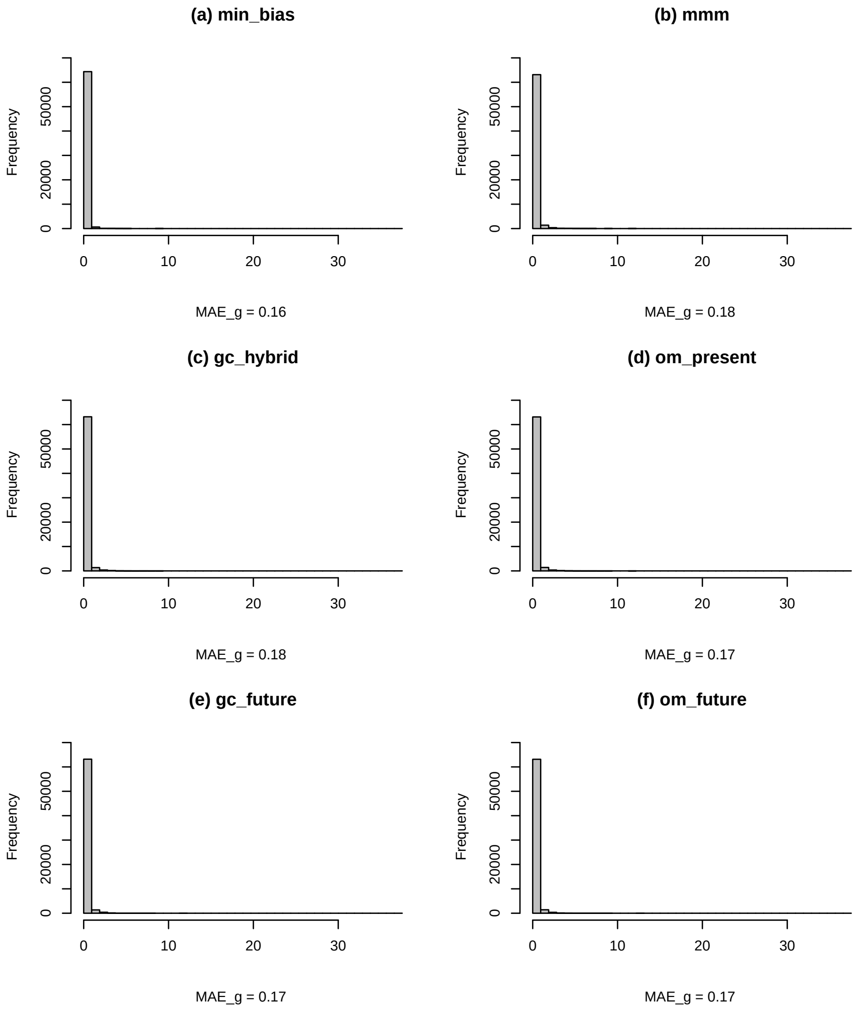

Fig. S6: Histograms of $MAE_g^{(p)}$ with respect to the reference ERA5 for the different combination approaches used to reconstruct the multi-decadal mean of PR over the period 1999-2019

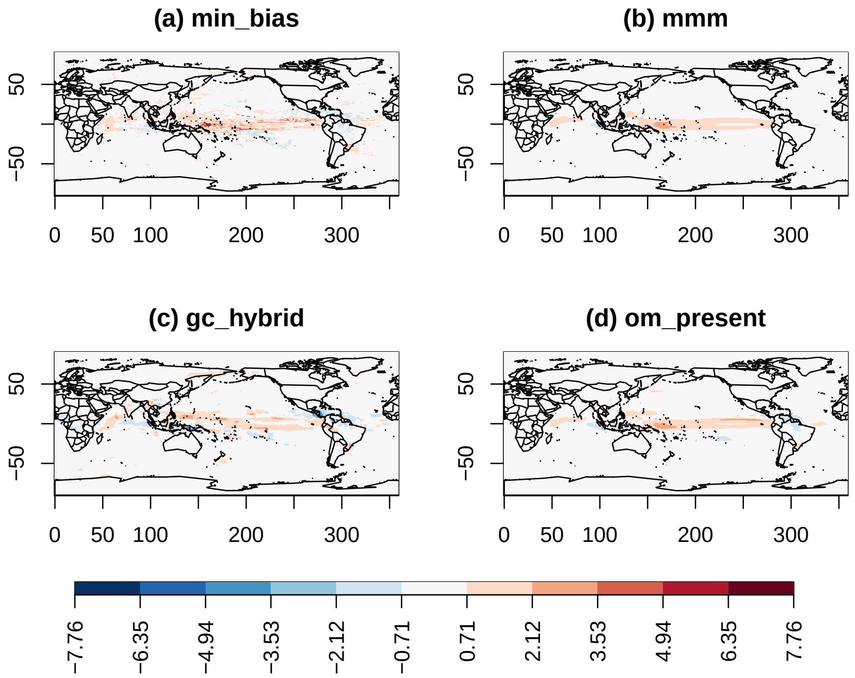

Fig. S7: Maps of the projected evolution of the multi-decadal mean of the variable PR, computed as the difference between periods 2071-2100 and 1999-2019. They are obtained for the ERA5 experiment with the following combination approaches: (a) min_bias, (b) mmm, (c) gc_hybrid, (d) om_present.

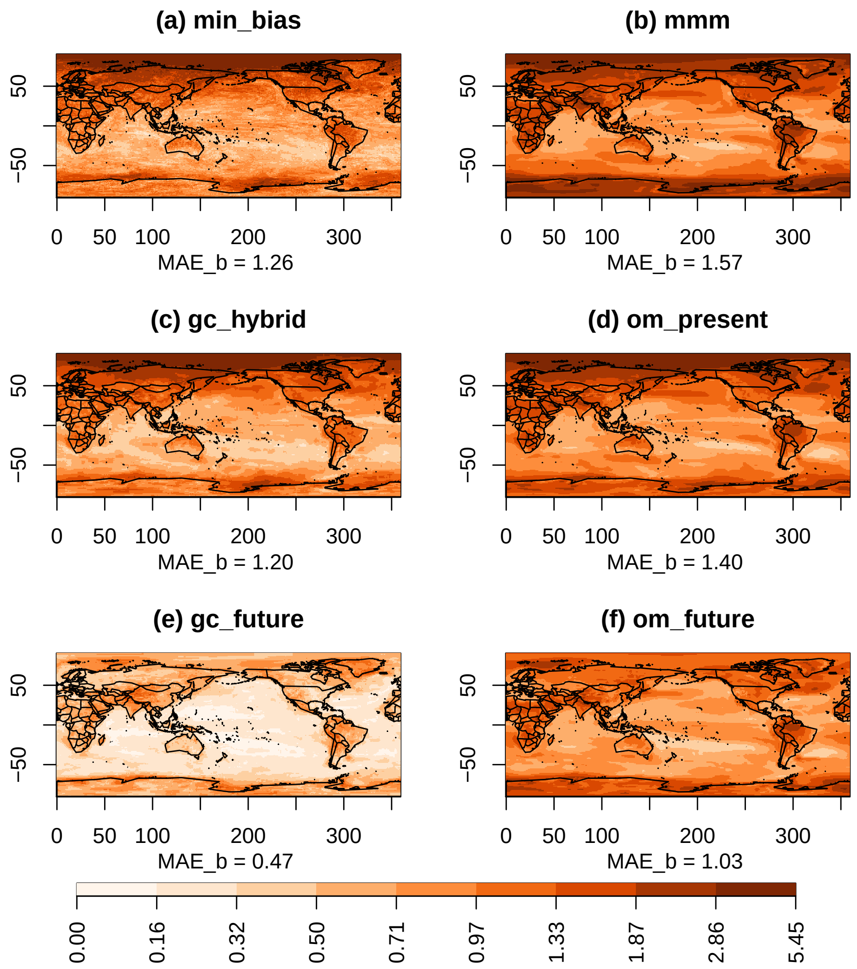

Fig. S8: Summary maps of the MAE_b of the variable TAS obtained for the period 2071-2100 and averaged over all references used in the perfect model experiment. Note that the color scale is not linear (arctangent scale).

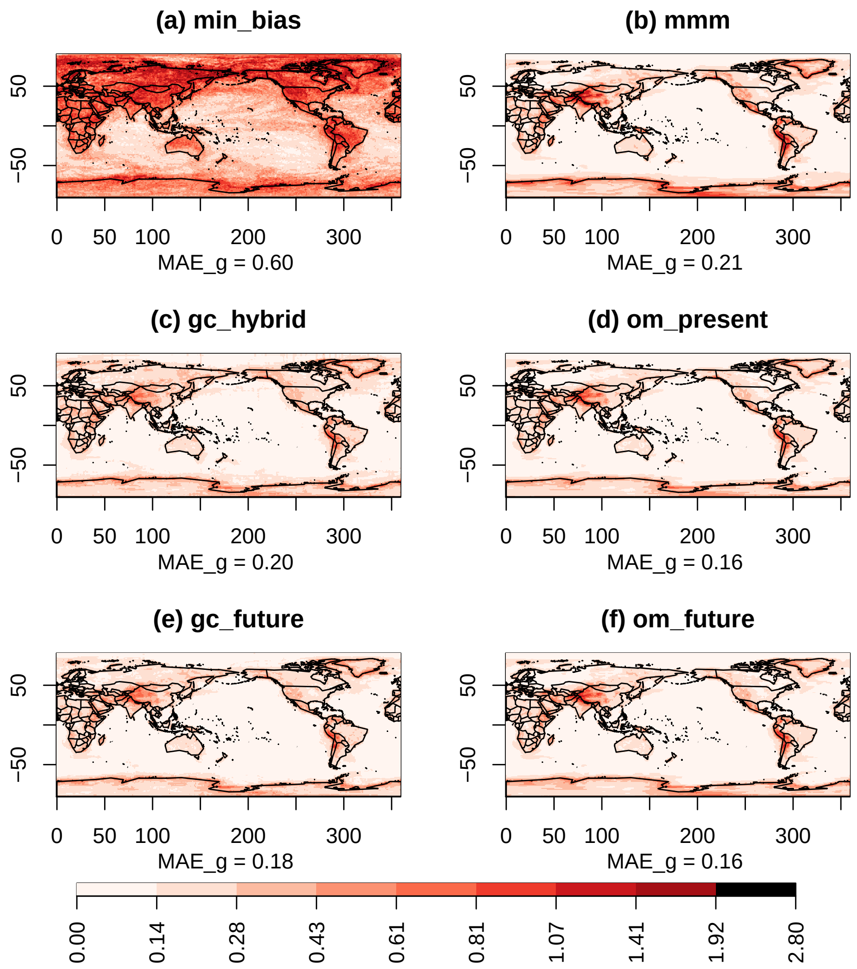

Fig. S9: Summary maps of the MAE_g of the variable TAS obtained for the period 2071-2100 and averaged over all references used in the perfect model experiment. Note that the color scale is not linear (arctangent scale).

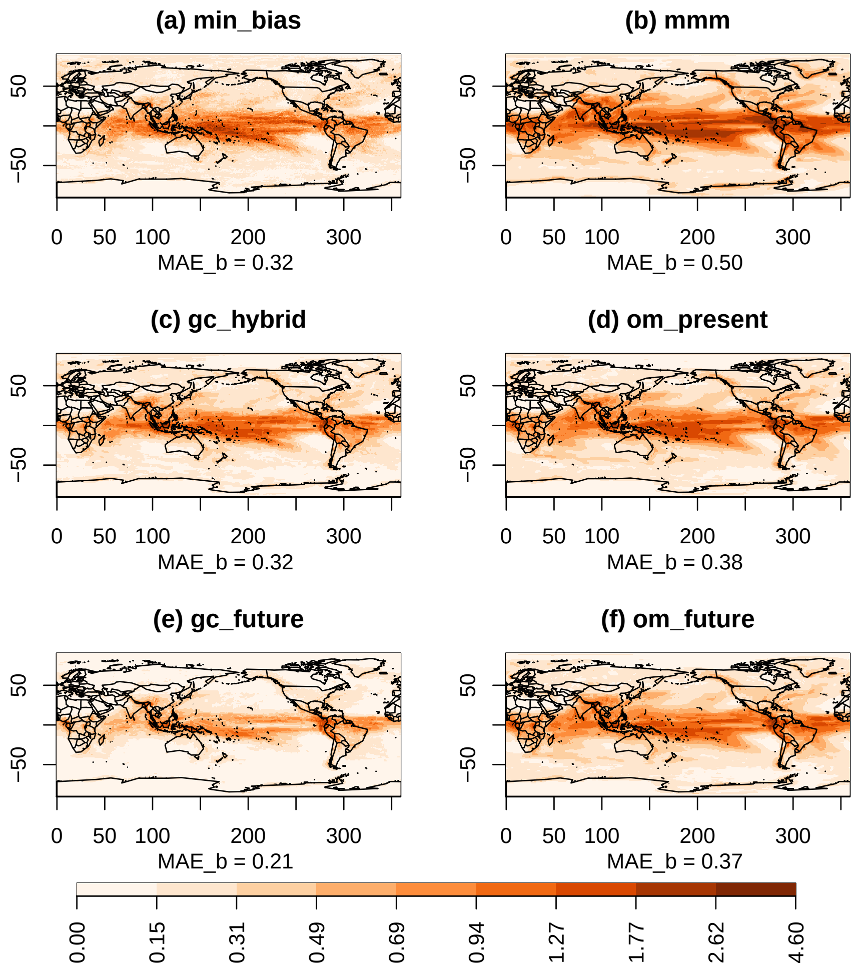

Fig. S10: Summary maps of the MAE_b of the variable PR obtained for the period 2071-2100 and averaged over all references used in the perfect model experiment. Note that the color scale is not linear (arctangent scale).

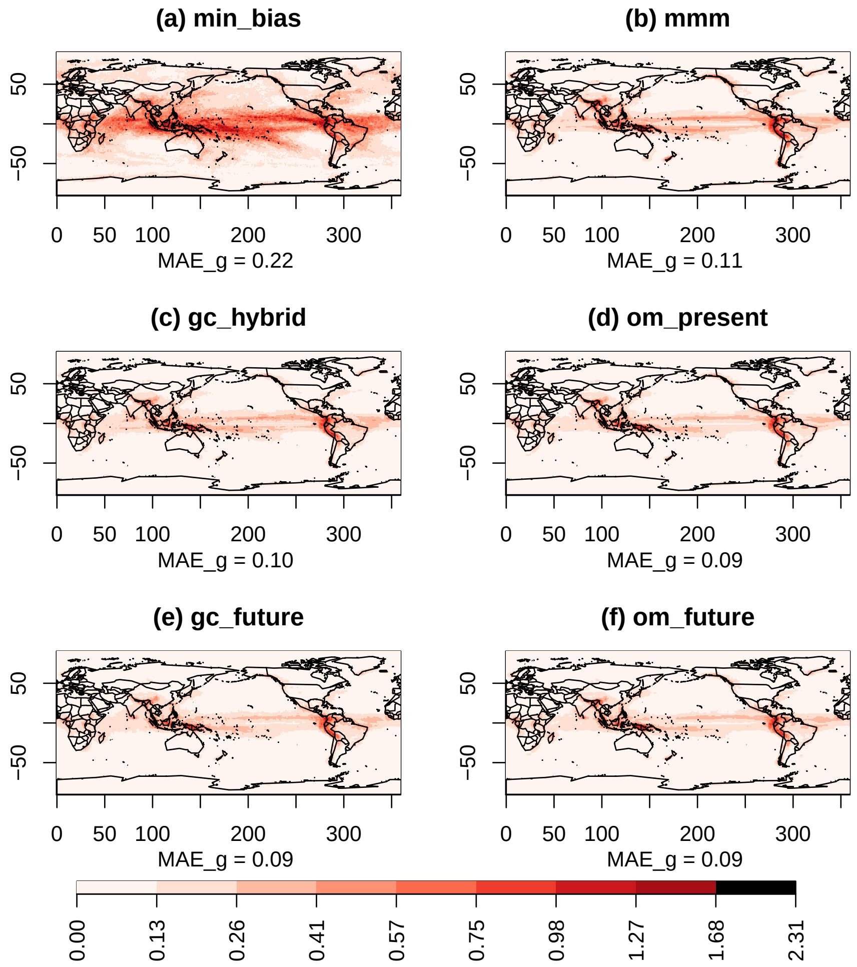

Fig. S11: Summary maps of the MAE_g of the variable PR obtained for the period 2071-2100 and averaged over all references used in the perfect model experiment. Note that the color scale is not linear (arctangent scale).
